# Patient Characteristics, Early Outcomes, and Implementation Lessons of Cervical Cancer Treatment Services in Rural Rwanda

**DOI:** 10.1200/JGO.18.00120

**Published:** 2018-11-30

**Authors:** Paul H. Park, Sonya Davey, Alexandra E. Fehr, John Butonzi, Cyprien Shyirambere, Vedaste Hategekimana, Jean Bosco Bigirimana, Ryan Borg, Regis Uwizeye, Neo Tapela, Lawrence N. Shulman, Thomas Randall, Egide Mpanumusingo, Tharcisse Mpunga

**Affiliations:** **Paul H. Park**, **Alexandra E. Fehr**, **Cyprien Shyirambere**, **Jean Bosco Bigirimana**, **Ryan Borg**, **Regis Uwizeye**, and **Egide Mpanumusingo**, Partners In Health/Inshuti Mu Buzima, Rwinkwavu; **John Butonzi**, **Vedaste Hategekimana**, and **Tharcisse Mpunga**, Butaro District Hospital, Rwanda Ministry of Health, Butaro, Rwanda; **Sonya Davey** and **Lawrence N. Shulman**, Abramson Cancer Center, University of Pennsylvania, Philadelphia, PA; **Paul H. Park** and **Neo Tapela**, Brigham and Women’s Hospital; **Paul H. Park**, Harvard Medical School; **Thomas Randall**, Harvard Medical School, and Massachusetts General Hospital, Boston, MA; and **Neo Tapela**, Oxford University, Oxford, United Kingdom.

## Abstract

**Purpose:**

Low- and middle-income countries account for 86% of all cervical cancer cases and 88% of cervical cancer mortality globally. Successful management of cervical cancer requires resources that are scarce in sub-Saharan Africa, especially in rural settings. Here, we describe the early clinical outcomes and implementation lessons learned from the Rwanda Ministry of Health’s first national cancer referral center, the Butaro Cancer Center of Excellence (BCCOE). We hypothesize that those patients presenting at earlier stage and receiving treatment will have higher rates of being alive.

**Methods:**

The implementation of cervical cancer services included developing partnerships, clinical protocols, pathology services, and tools for monitoring and evaluation. We conducted a retrospective study of patients with cervical cancer who presented at BCCOE between July 1, 2012, and June 30, 2015. Data were collected from the electronic medical record system and by manually reviewing medical records. Descriptive, bivariable and multivariable statistical analyses were conducted to describe patient demographics, disease profiles, treatment, and clinical outcomes.

**Results:**

In all, 373 patients met the study inclusion criteria. The median age was 53 years (interquartile rage, 45 to 60 years), and 98% were residents of Rwanda. Eighty-nine percent of patients had a documented disease stage: 3% were stage I, 48% were stage II, 29% were stage III, and 8% were stage IV at presentation. Fifty percent of patients were planned to be treated with a curative intent, and 54% were referred to chemoradiotherapy in Uganda. Forty percent of patients who received chemoradiotherapy were in remission. Overall, 25% were lost to follow-up.

**Conclusion:**

BCCOE illustrates the feasibility and challenges of implementing effective cervical cancer treatment services in a rural setting in a low-income country.

## INTRODUCTION

The majority of cancer morbidity and mortality occurs in low- and middle-income countries (LMICs), which account for 56% of new cancer cases, 62% of cancer deaths, and 69% of cancer-caused disability-adjusted life years globally.^1^ The World Health Organization’s Global Cancer Database (GLOBO-CAN 2012)^2^ estimated that there were 645,000 new cases of cancer and 456,000 cancer-related deaths in sub-Saharan Africa (SSA) in 2012. Although the incidence and mortality from cancer in SSA is expected to double by 2030,^2^ only 0.3% of global cancer expenditures are delivered in this region.^3^ Cervical cancer is no exception to this global trend. LMICs bear 86% of all global cervical cancer cases and 88% of global cervical cancer mortality.^4^ SSA specifically has the highest morbidity and mortality rates globally. Despite having 9% of the world’s female population older than 15 years of age, SSA is home to 18% of cervical cancer deaths worldwide.^5^

The gap in morbidity and mortality between LMICs and high-income countries (HICs) is, in part, derived from the limited availability of screening and prevention programs. In 2012, only six LMICs had national immunization programs for human papillomavirus (HPV) vaccination,^6^ and only a minority of women in LMICs are screened for cervical cancer in their lifetime.^7,8^ This lack of early detection programs then drives the well-documented disparities in stage of presentation between LMICs and HICs across all cancers, including cervical cancer.^9,10^ Studies from SSA hospitals that have treatment programs for invasive cervical cancer demonstrate that between 47% and 89% of patients initially present with an advanced stage (stage IIB or higher).^11-17^ In comparison, in the United States, 40% of patients with cervical cancer present at an advanced stage.^18^ These data highlight the need for improved access to diagnosis and staging programs in SSA.

Access to cervical cancer treatment is limited across SSA. For example, based upon guidelines from the International Atomic Energy Agency,^19^ SSA met only 18% of the radiotherapy needs for its population size in 2010.^3,20^ Studies from Nigeria, Tanzania, Uganda, and Zimbabwe indicate that only 34% to 67% of women diagnosed with cervical cancer receive treatment.^13,21-24^ Retention to manage chronic disease in SSA is also a challenge. Across chronic disease clinics of low-income countries (LICs) in SSA, studies have commonly shown lost-to-follow-up (LTFU) rates to be greater than 30%.^25,26^ In addition, SSA treatment program outcomes and implementation experiences and challenges are not well documented.

In July 2012, the Rwandan Ministry of Health (RMOH) opened the Butaro Cancer Center of Excellence (BCCOE) as the country’s first facility for delivering health care services for a broad range of cancers.^27,28^ BCCOE, located within the Butaro District Hospital in rural Rwanda, is a public-sector RMOH facility supported by Partners In Health/Inshuti Mu Buzima (PIH/IMB), the Dana-Farber/Brigham and Women’s Cancer Center (DFBWCC), and the University of Pennsylvania. Patients present to BCCOE as either self-referral or referrals from Rwandan or other East and Central African referral and district hospitals, thus making BCCOE the national referral hospital for oncology.

Although LMICs are often grouped together, LICs generally have far fewer resources for treatment of invasive cancers than do middle-income countries.^29^ BCCOE’s implementation experience on cervical cancer, the second most common diagnosis at BCCOE,^30^ provides a model for quality cancer diagnosis, staging, and referred treatment in a severely resource-limited setting. In this retrospective descriptive study, we describe the successes and challenges of care delivery by describing the patient population, clinical presentation, treatment received, early outcomes, and implementation experience of cervical cancer management at BCCOE. For early clinical outcomes, we hypothesize that the last status (eg, alive, deceased, LTFU) will vary significantly by stage at presentation and whether chemoradiotherapy treatment was received. Specifically, those presenting at earlier stage and receiving treatment will have higher rates of survival.

## METHODS

### Service Implementation

The implementation of the entire BCCOE delivery model is well described elsewhere.^28,30,31^ Briefly, establishing partnerships was key to the implementation of cancer care at BCCOE.^27,32^ To ensure guidance from cancer care experts, RMOH and PIH requested support from DFBWCC, whose staff committed to providing critical support in areas of remote and on-site oncology training and mentorship to physicians and nurses, protocol development, focused development of high-quality pathology laboratory and remote telepathology capacity, establishment of a robust clinical database for monitoring and evaluation purposes, and dissemination of outcomes through research. This collective team began by designing an implementation work plan, which focused on the vision, distribution of responsibilities, communication structures, and establishment of essential oncology-specific health system building blocks. Early on, the establishment of national clinical protocols provided clear directive for procuring essential medicines, equipment, and consumables. Regular conference calls and on-site visits were essential to design, deliver, and maintain the above-mentioned areas of support.

Designing a cervical cancer treatment program requires a broad range of services. Cervical cancer is a malignancy caused by a persistent high-risk oncogenic HPV infection leading to dysplasia at the transformation zone of the cervix. Ultimately, this can cause squamous cell carcinoma or adenocarcinoma.^33^ Definitive diagnosis requires biopsy, and follow-up staging is accomplished by physical examination and radiographic imaging. Generally, treatment for cervical cancer includes surgical resection at early stages and chemoradiotherapy at advanced stages.^34^

Cervical cancer treatment services were implemented at BCCOE in 2012 with cervical cancer being one of the initial priority cancers to be treated. Treatment protocol included diagnosis based on classic physical examination findings for locally advanced cervical cancer. The lack of a requirement for pathologic confirmation was standard of care across multiple SSA countries at the time.^35^ The development of biopsy and pathology capabilities was part of the initial implementation plan for improving care. The current national protocol requires biopsy confirmation for diagnosis, followed by clinical and radiologic staging according to the nternational Federation of Gynecology and Obstetrics (FIGO) staging system. Once patients with cervical cancer are diagnosed and staged, treatment options include (1) referral to Kigali for surgical resection or hysterectomy (stage IA to IIA), (2) referral to the Uganda Cancer Institute (UCI) in Kampala, Uganda, for concurrent chemoradiotherapy (stage IB to III), or (3) palliative care. Most palliative care patients were transferred to their local district hospital for ongoing follow-up. For those patients in remission, BCCOE provided protocol-based follow-up services.

Once biopsies were performed at BCCOE, the pathologic diagnosis for patients with cervical cancer initially required sending all specimens to the DFBWCC in Boston, MA.^32^ Once the specimens DFBWCC received the specimens, pathologists would generate the final results and send them electronically to BCCOE after approximately 4 to 8 weeks from specimen collection. In 2014, telepathology services for all biopsy specimens were implemented at BCCOE through a partnership with pathologists at DFBWCC. The turnaround time was significantly shortened as a result.^36^ Radiographic staging at BCCOE is limited to ultrasound and plain film radiography.

The provision of radiotherapy services at UCI required designing and implementing a comprehensive standard operating procedure document that provided details regarding patient inclusion criteria, transportation of patients to UCI, care delivery at UCI, and communication of treatment received. Because resources were limited, a committee at BCCOE selected 15 patients to receive radiotherapy each month. Patients were prioritized on the basis of best estimates of prognosis, functional status, and an estimate of the net benefit of radiotherapy. A nurse accompanied all patients during the 9-hour ground travel to and from UCI and to initial orientation to their accommodations and the medical facilities. Once qualified patients with cervical cancer arrived at UCI, they received cisplatin chemotherapy per protocol, external beam radiation therapy at a dose of 50 Gy in 25 fractions, and brachytherapy at total dose of 30 Gy over six sessions.

The establishment of paper clinical forms and their analogous electronic medical record (EMR) forms (using the OpenMRS platform) greatly improved the reports used to monitor and evaluate cervical cancer indicators.^31^ Although some data were directly entered into the EMR by the clinician, a majority of the data were entered on paper forms and were subsequently transcribed into the EMR by data officers on a weekly basis. BCCOE staff could then ask EMR programmers and coordinators to produce routine indicator reports. This data collection and reporting process allowed for valuable monitoring and evaluation as well as research to inform areas for future growth. BCCOE staff routinely reviewed data through several forums, including monthly research meetings and ad hoc quality improvement meetings. Participants included EMR, monitoring and evaluation, research, clinical, and administrative leadership staff.

### Study Design

This is a retrospective descriptive study of patients with cervical cancer from BCCOE. Patients were included in the study if they had a cervical cancer diagnosis and an intake date from BCCOE between July 1, 2012, and June 30, 2015. Patient information was included up to the date of data extraction (August 10, 2016). OpenMRS was used to identify all patient records that met the inclusion criteria and to extract relevant patient data. When necessary, additional patient data were added via manual review of medical records. Collected data included patient demographics, clinical history, disease profile, treatment details, and early clinical outcomes.

Clinical outcomes were based on the last known status of the patient at the time of data extraction and were collapsed into six categories: (1) patients who were alive and completed treatment; (2) patients who were alive and continuing treatment; (3) patients who were deceased; (4) patients who were lost to follow-up, defined as not having a clinical encounter for 6 months or more; (5) patients referred to palliative care; and (6) all other possible outcomes. Treatment in this case includes chemoradiation as well as surgery. Other possible outcomes included transfer to another facility or declining further treatment. Because these outcomes were still early, the median number of months from date of diagnosis to date of last visit was calculated to provide the follow-up time period for the six clinical outcomes.

### Data Analysis

Data analysis was conducted using Microsoft Excel and STATA v. 13 (STATA, College Station, TX). Descriptive statistics were used to demonstrate patient demographic information, disease profiles, treatment details, and clinical outcomes. Pearson’s χ^2^ bivariate analysis was used to determine whether the patient received chemoradiotherapy. A Kruskal-Wallis test was used to compare the median number of months from date of diagnosis to date of last visit across disease stage at presentation. This study was approved by the Rwanda National Ethics Committee and the Inshuti Mu Buzima Research Committee.

## RESULTS

### Patient Profile

In all, 373 patients with cervical cancer met the inclusion criteria. The median age was 53 years (interquartile range [IQR], 45 to 60 years). Twenty-eight percent of included patients came from the Northern Province where BCCOE is located. Sixty-four percent of patients presented with an Eastern Cooperative Oncology Group (ECOG) performance status of less than 2, and 29% were laboratory-confirmed HIV positive. Thirteen percent of patients had a hysterectomy before presenting at BCCOE (Table 1).

**Table 1:**
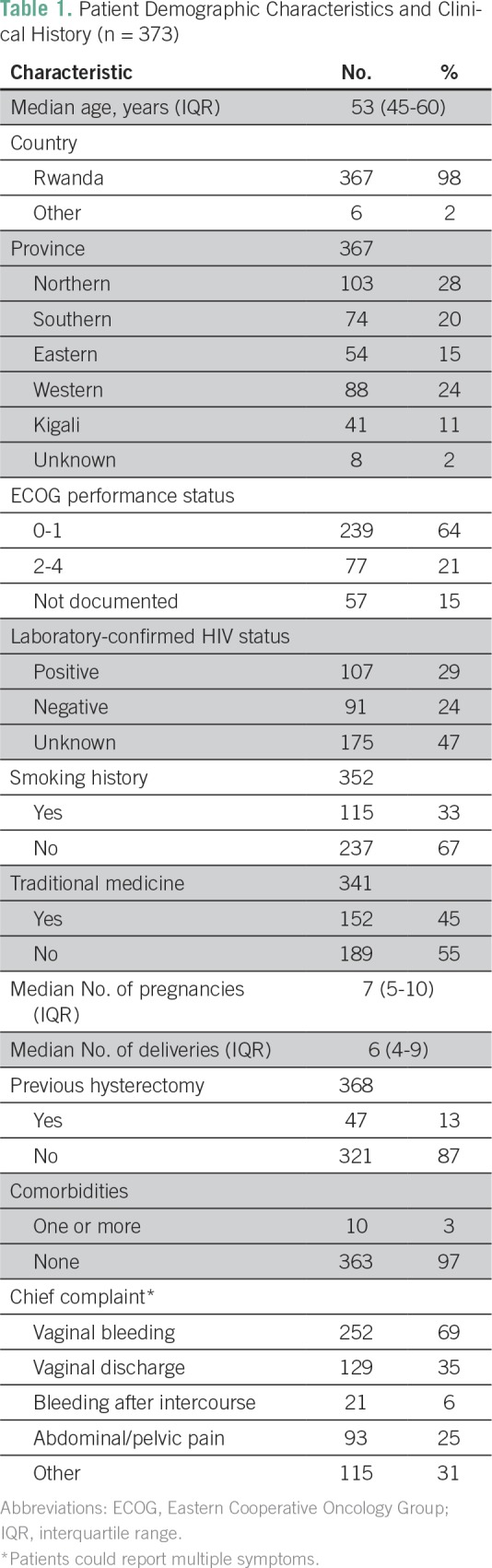
Patient Demographic Characteristics and Clinical History (n = 373)

### Disease Profile and Treatment

Only 68% percent of patients had a documented pathologically confirmed diagnosis, and for staging purposes, 52% of patients did not have any documented radiology results. Eleven percent of patients did not have a documented stage; in addition, 3% were stage I, 48% were stage II, 29% were stage III, and 8% were stage IV at presentation (Table 2). Treatment with curative intent, as opposed to palliative or undecided, was planned for 50% of the patients, and nearly all (96%) of these patients were referred for chemoradiotherapy. Among those referred, 80% received chemoradiotherapy. Chemoradiotherapy patients arrived at UCI a median of 1.3 months (IQR, 0.8 to 2.6 months) after diagnosis; they attended a follow-up appointment at BCCOE at a median time of 3.4 months (IQR, 3.2 to 3.8 months) after arriving for chemoradiotherapy treatment (Tables 2 and 3).

**Table 2:**
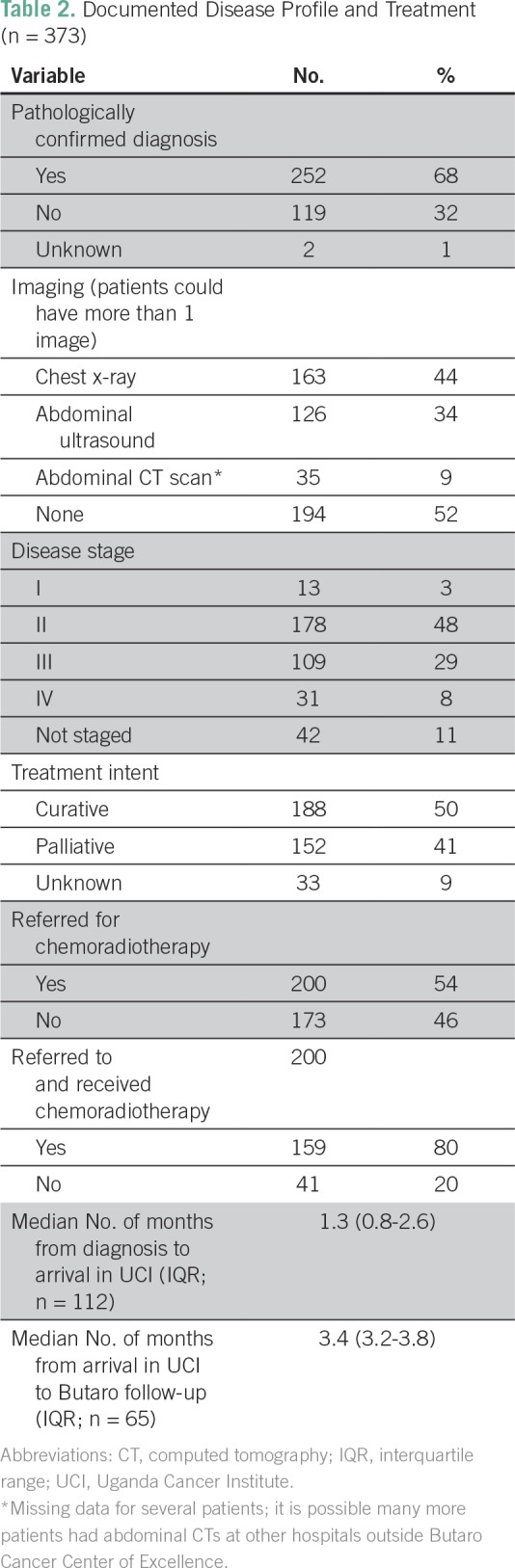
Documented Disease Profile and Treatment (n = 373)

**Table 3:**
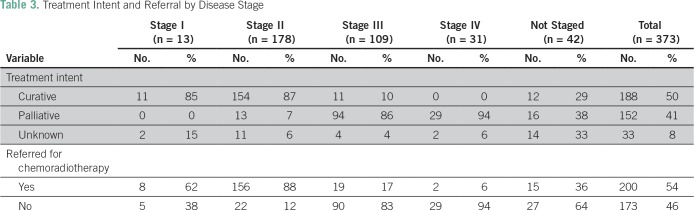
Treatment Intent and Referral by Disease Stage

### Outcomes

**Fig 1:**
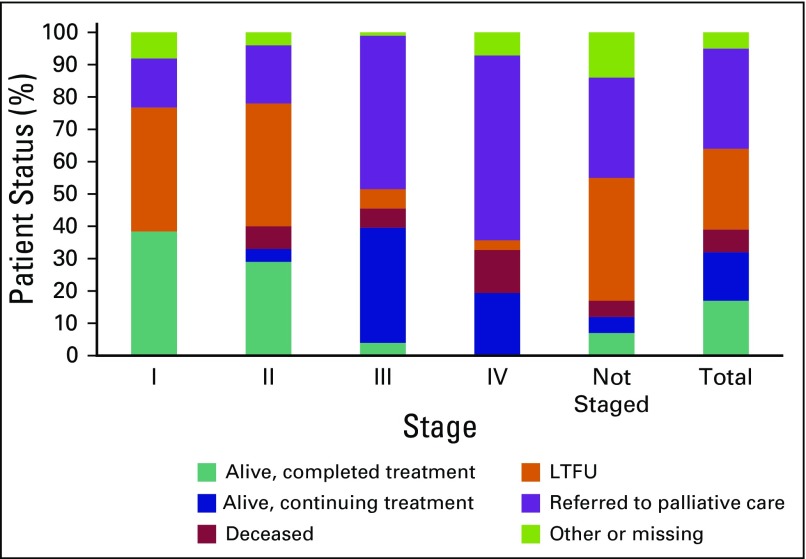
Percent patient status by disease stage at presentation. LTFU, lost to follow-up.

**Fig 2:**
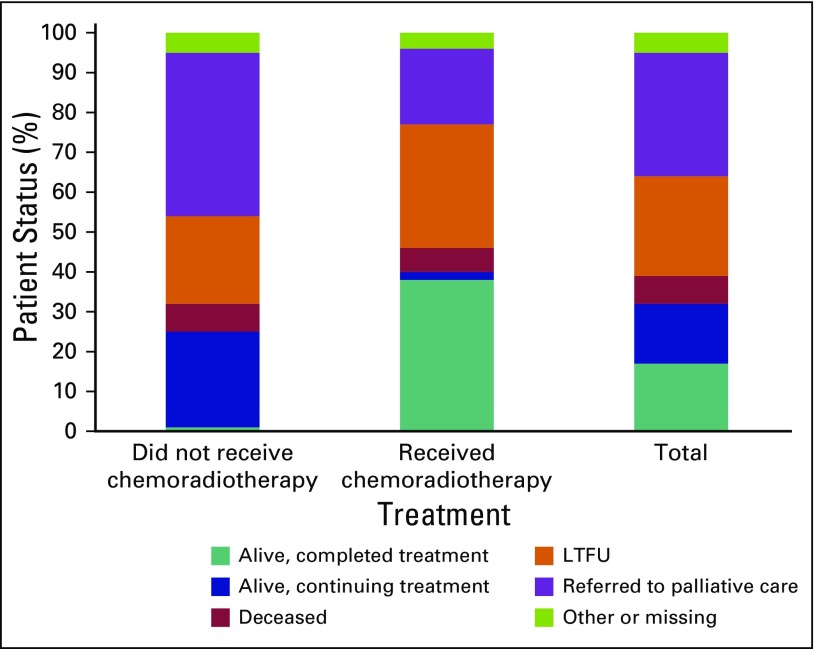
Percent patient status for those receiving chemoradiotherapy or not. LTFU, lost to follow-up.

Overall, 17% of patients completed treatment with no evidence of recurrence at last status (Table 4). This included 38% of stage I, 29% of stage II, 4% of stage III, and 0% of stage IV patients. The overall rate of LTFU was 25%, with a median follow-up time of 3.8 months (IQR, 0 to 20.8 months) from date of diagnosis to date of last visit (Table 4 and Fig 1). Patients who received chemoradiotherapy had significantly better outcomes than those who did not receive any chemoradiotherapy (*P* < .001): 38% of referred patients who received chemoradiotherapy were alive and had completed treatment at the time of data extraction compared with only 1% of those who did not (Table 4 and Fig 2).

**Table 4:**
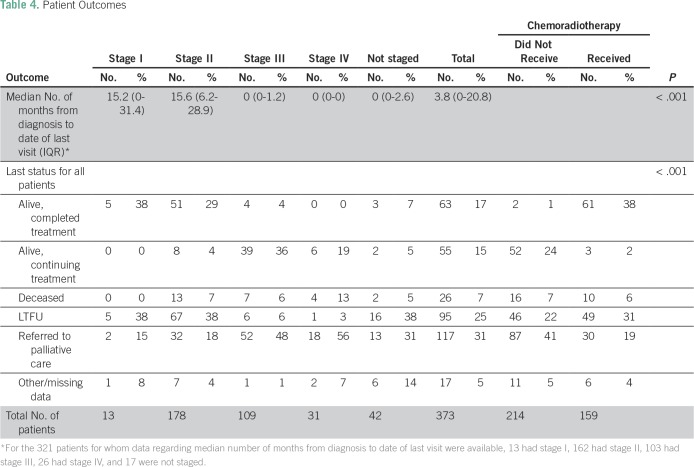
Patient Outcomes

## DISCUSSION

The establishment of cervical cancer treatment services at BCCOE required methodical, stepwise implementation procedures to overcome barriers inherent to SSA and produced promising early outcomes. Thirty-eight percent of patients with cervical cancer who enrolled and received chemoradiotherapy during the first 3 years of the program are in remission and have not been LTFU. Although this value is not as high as outcomes found in HICs, it illustrates the capacity to save many lives. Unlike facilities in HICs, BCCOE accomplished this outcome even though it did not have a domestic radiotherapy facility and it had a higher proportion of late-stage presentations. Late presentations explain, in part, the higher mean age of presentation compared with that of HICs, which is typically younger than 50 years of age.^37^ We also suspect that with nearly half of patients having previously engaged with traditional healers, there is persistent limited knowledge in the community regarding the value of early-stage presentation for cancer care.

The challenges of late-stage presentation and limited resources, which prevent more patients from receiving chemoradiotherapy, are the most critical barriers to better patient outcomes. In our setting, patients with stage III or IV disease often do not qualify for radiotherapy because of the methods in the protocols for prioritizing patients, and those patients face a worse prognosis after treatment when such treatment is available. Thus, more than three quarters of these late-stage patients are given palliative care only. To combat this challenge, the RMOH is currently piloting health center–focused visual inspection with acetic acid (VIA)–based screening and HPV testing by polymerase chain reaction in addition to existing national HPV vaccination efforts.^38,39^

The resource limitation surrounding access to radiotherapy is especially critical for patients with stage III cervical cancer, of whom few are referred for curative treatment. In HICs, cure rates for patients with stage IIIa cancer are as high as 60% at 2 years and 40% at 5 years.^40^ Our experience demonstrates that it is feasible to cure many patients with invasive cervical cancer through international transfer for chemoradiotherapy. However, BCCOE faced many logistical and financial challenges while trying to complete international transfers. Fortunately, the Government of Rwanda has recently opened its first radiotherapy center.

The LTFU rate was high (25%), but not outside expectations when compared with other chronic diseases in similar settings.^25,26^ Likely contributors include the long travel distances required for most patients.^41^ As expected, late-stage patients had a lower LTFU rate because they are oftentimes quickly transferred to the nearest health facility for palliative care. In response to LTFU challenges, BCCOE has implemented a call-back log form and protocol, whereby the EMR produces a missed visit report of all patients who do not present to the hospital during the expected week. Nurses then systematically call such patients to encourage them to return for care. Those patients who do not answer the phone after three attempts over a 6-month period are then deemed LTFU and are removed from the program. Given the unique socioeconomic barriers to accessing care in our setting, it would be of value to conduct a comparative study that assesses the impact of the call-back system in relation to other facilities without this intervention. 

### Limitations

The patient status values are not based on an extensive period of follow-up. The median follow-up time is 3.8 months (IQR, 0 to 20.8 months) for all patients and 15.6 months (IQR, 6.2 to 28.9 months) for stage II patients who were the primary group receiving prolonged treatment without planned transfers out for palliative care. Future studies with longer follow-up time may illustrate a different distribution across the clinical statuses. In addition, the patient demographics did not include whether the patients came from a rural or urban setting. Such data would have allowed valuable analysis of stage of presentation and patient status by urban versus rural home location. Finally, this study does not include other implementation science outcomes, such as fidelity to clinical guidelines, cost, or staff retention.

Our comorbidities, diagnosis, and staging results highlight data quality challenges inherent to using routinely collected EMR data for research as well as additional shortcomings unique to resource-limited settings. First, comorbidities seem to be under-reported, given the higher median age. Future studies should ensure that details regarding this variable are included. Second, initially, pathologically confirmed diagnosis was not standard of care across the region because of limited resources. However, during the study window, BCCOE elected to improve their standard of care to pathologic confirmation by instituting not only cervical biopsy capacity but also telepathology services. Given these changes and poor documentation, we cannot accurately quantify the number of patients treated without a biopsy diagnosis. In terms of documentation on treatment with radiology, limited results (52%) illustrate the significant challenges for clinicians in adhering to the staging protocol and maintaining complete records of highly complex patient care. In part, this may be because paper forms currently lack streamlined, guided features such as tick boxes and detailed prompts. Furthermore, data officers may not be consistently transcribing all necessary data into the EMR. More tailored paper and electronic forms are being designed and clinicians are being trained on how to properly fill in a form. The RMOH has the long-term goal of having clinicians enter data directly into the EMR, which could potentially increase opportunities to attain more complete documentation and help clinicians with decision making.

The cervical cancer services at the BCCOE illustrate the feasibility of successfully treating patients with cervical cancer in an LIC despite not having domestic radiotherapy services. As expected, our cure rates are not as high as those of HICs; however, we have been able to show that many lives can be saved with our services, particularly for patients who present at an early stage. We still need improved implementation design and research on early detection and retention to care. Referring patients for radiotherapy within Rwanda will likely decrease both costs and programmatic barriers, which we hope will increase the volume and types of patients who can be treated and decrease the financial and social burdens on Rwandans who need radiotherapy. The cervical cancer services at BCCOE provide a model of care that can overcome barriers inherent to resource-poor settings and show that it is possible to close the access and outcomes gaps between HICs and LICs.
